# Factors related to dietary habits, energy drink consumption, and physical activity in marginalized Palestinian schools: A cross-sectional study

**DOI:** 10.34172/hpp.2021.42

**Published:** 2021-08-18

**Authors:** Sami Amer, Elham Kateeb

**Affiliations:** ^1^MIT Media Lab, Massachusetts Institute of Technology, 77 Massachusetts Avenue Cambridge, MA 02139, USA; ^2^Oral Health Research and Promotion Unit, Faculty of Dentistry, Al-Quds University, Jerusalem, State of Palestine

**Keywords:** Diet habits, Physical activity, Body mass index, Energy drinks, Adolescents health

## Abstract

**Background:** The current study assessed different dietary habits, energy drinks intake, body mass index (BMI) and physical activity and associated factors among Palestinian adolescents attending marginalized schools.

**Methods:** A cross-sectional study targeted a random sample of 1480 students in the sixth andninth grades attending 20 marginalized public schools in the West Bank area of the occupied Palestinian Territories (oPt). Students were interviewed in-person by trained senior dental students about their dietary habits, physical activity, fathers’ employment and mothers’ level of education. Weight and height were measured, and BMI percentile was calculated. Descriptive statistics were generated for the study’s main variables and the dependent variables were compared by grade, gender, mothers’ level of education and father’s employment.

** Results:** A total of 1282 students (98% response) completed the questionnaire. Of them, 6% were ‘underweight’ (fifth percentile or under) and 34% were ‘overweight’ or ‘obese’ (85thpercentile or over). Ninth graders had more added sugar than 6th graders (P=0.002), less frequent milk consumption (P<0.001), more frequent energy drink consumption (P=0.001),and less physical activity (P<0.0001). Frequency of carbonated and sweetened drink consumption was associated with being overweight or obese (P=0.016, P=0.001). Frequency of carbonated drinks consumption was higher among children of mothers with a high school level of education or less (P<0.001). In addition, children of mothers educated to high school level or below were associated with being underweight (P=0.05).

**Conclusion:** Dietary habits and physical activity get worse between the ages of 12 and 15. Mothers’ level of education is an important factor in being overweight or underweight. Energy drink consumption among boys and milk consumption among girls are two challenges that need to be addressed urgently and adequately.

## Introduction

Obesity was classified by the World Health Organization (WHO) as one of the public health threats in the 21^st^ century. Different physical, social and psychological negative outcomes were associated with obesity. Cardiovascular disorders,^1^ Type II diabetes,^2^ and pulmonary,^3^ hepatic,^4^ renal,^5^ and musculo-skeletal^6^ complications, certain types of cancers^7,8^ are common consequences of obesity. In addition, being overweight or obese among schoolchildren was associated with diminished quality of life,^9,10^ risk of adverse emotional states,^11,12^ negative stereotyping,^13^ bullying and social isolation.^14^

Several factors contribute to globally accelerated trends of overweight and obesity in children and adolescents. These include genetic, metabolic, behavioral, environmental, socio-cultural, and socioeconomic factors. Although increased body weight is a multi-factorial process, unfavorable dietary habits and/or inadequate physical activity can be considered two main mediators of this condition.^15^

Key characteristics of adolescents’ dietary habits usually include snacking, skipping breakfast, dieting and adoption of specific diets.^16^ In addition, previous literature found that quality of diet and physical activity among adolescents is usually associated with their mothers’ educational levels and family incomes.^17-19^

Moreover, the current data on obesity transition suggest that a representative 77.5% of the world’s population usually goes from high prevalence of obesity with marked variation among age groups and gender to less prevalent obesity with more equal distribution among age and gender.^20^

All previous factors are heavily influenced by cultural and social norms within a particular society, which makes studying dietary habits, physical activity and their related influencers an important area of research in order to understand social determinants of health and predictors of disease within a certain population.^21^In addition to previous factors, the WHO’s Commission on Social Determinants of Health indicates that political conflict can hinder access to health facilities and is considered detrimental to health.^22^

Previous data on dietary habits and physical activities among Palestinian adolescents were usually carried out in particular geographic areas, mainly Area A and B, with no focus on schoolchildren living in disadvantaged areas, found in Area C.

In the Occupied Palestinian Territories (oPt), many areas that are under conflict and political unrest suffer from challenging health, social services and interventions delivery due to the socio-political and administrative context, and restrictions in movement and transportation. A clear example of these conditions is found in Area C in the oPt.^23^ The Oslo Accords, in order to achieve resolution to conflict in the oPt for a transitional period, divided the oPt into three zones: Area A, consisting of only 3% of the land, “where the Palestinian National Authority (PA) assumed control of all civilian administration, including health and security”^23^;Area B, consisting of 27% of the land, “where the PA has civilian authority, but shares security responsibility with Israel”^23^; and Area C, “where the PA has no control over the remaining 70% of land”. ^23^

Therefore, the current study assessed different dietary habits, energy drinks intake, body mass index (BMI) and physical activity and associated factors among Palestinian adolescents attending marginalized schools in the West Bank area of the Palestinian territories, mainly Area C schools. Schools were categorized as marginalized by the School Support Program (SSP) implemented by the American-Middle East Educational and Training Services, Inc (ANIDEAST)/Palestine.

## Materials and Methods

This is a cross-sectional study that was conducted in 20 marginalized schools in the West Bank area of oPt. The SSP is a United States Agency for International Development (USAID)-funded initiative launched in 2013 and executed by AMIDEAST/Palestine in partnership with the Save the Children Organization.^24^ The SSP, in collaboration with the Palestinian Ministry of Education (MOE), identified the 50 most educationally marginalized schools out of 1700 schools in the West Bank area of the oPt. Selection criteria were based on poverty, educational achievement, drop-out rates, infrastructure needs, building status, limited access, economic marginalization, and under-qualified teachers. ^24^

**Figure 1:**
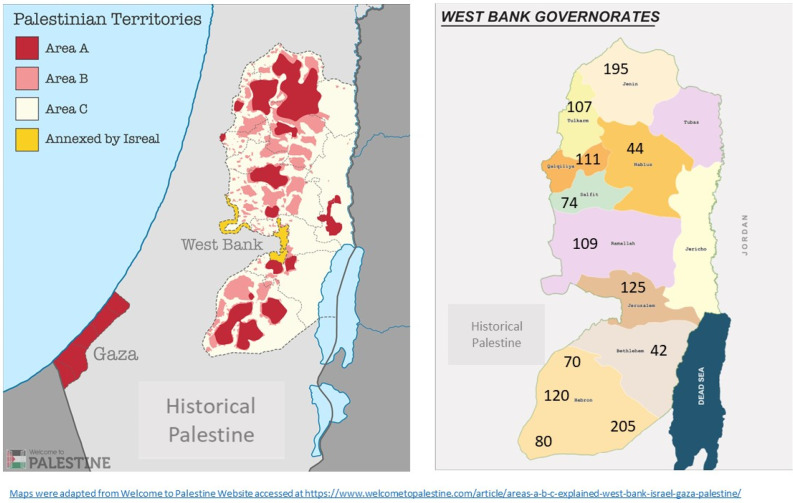
Demonstration of school districts, number of students in each district and areas A,B and C according to Oslo Accord. The map on the right shows school districts included in the sample and number of students in each school district. The map on the left shows the distribution of Area A,B and C according to Oslo Accord

Most of the schools included in the SSP are in Area C according to the Oslo Accord.^23^ Maps of school districts included in the sample and Areas A, B and C according to the Oslo Accords are shown in Figure 1.

### Sampling technique

Out of the 50 SSP marginalized schools, a cluster stratified sampling technique was used to select representative schools for age, gender and education districts of the MOE. The target population in this study were students in sixth and ninth grades in the 50 SSP marginalized schools (n = 4688). The final selected 20 schools were representative of MOE educational districts for both genders and grade levels in the West Bank area of the oPt (n = 12 districts) which included 1480 students (the final sample was selected proportionally to the number of students in each district). Sample size calculation was carried out on our target population (n = 4688) using 95% confidence level and 3% margin of error. A minimum sample of 870 students was needed to ensure the accuracy of the results in the current study.

**Figure 2:**
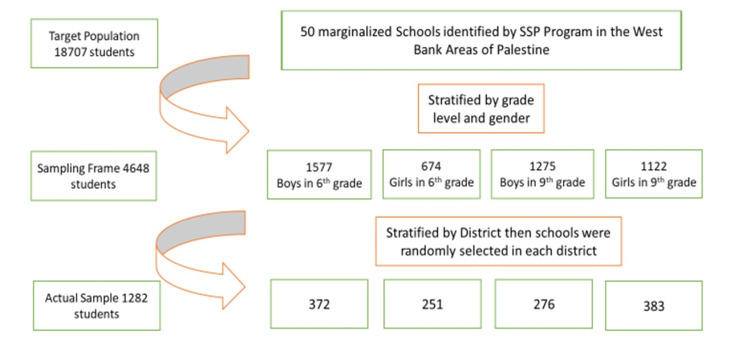
Flow chart about the stratified cluster sampling technique used in this study

All students in sixth and ninth grades in the sampled schools were recruited and consent forms were sent to students’ parents to approve students’ participation. Only healthy students with no systemic conditions were included in the final analysis. A flow chart detailing the sampling technique is shown in Figure 2.

### Dietary assessment

Students in the sixth and ninth grades were interviewed in-person by senior dental students as part of a bigger survey that addressed schoolchildren’s oral health status and dietary habits between January and December, 2018. Senior dental students were trained on how to use the structured survey in two-hour training sessions. Dietary habits questions used in the instrument were adapted after a published survey that was validated in the Palestinian community.^25^ The final draft of the questionnaire was additionally checked for content validity by six experts in the field of Public Health and Nutrition to check suitability of the questions to the population under study. Face validity was conducted to double check question clarity. This was done by pilot testing the survey with 30 students of the same ages as those in our target population sample. Questions were reviewed and modified according to the feedback obtained from the validation process.

Questions in this instrument covered specific demographic data such as gender, grade level, father’s employment status and mother’s education level. Dietary habit questions included inquiries about number of meals, snacks and frequency of some dietary intake such as milk, fruits and vegetables, added sugars and energy drinks, among others. A detailed list of the dietary assessment items are presented in Tables 1 and 2. In addition, a question was included that assessed amount of physical activity per week. Physical activity was dichotomized to 5 or more times per week and less than 5 times per week based on a methodology used in a nutritional national survey of Palestinian children.^26^

**Table 1:** Dietary habits among sixth and ninth graders attending SSP marginalized schools, n=1280

**Diet Habits**	**6th Grade n=623** **Mean±SD**	**9th Grade n=659** **Mean±SD**	***P*** ** Value**
Sugar (spoons/day)	2.3 ± 1.8	2.7 ± 3.3	.002**
Milk (cups/week)	1.0*(0-1)	0.0*(0-1)	.001***
Sweets and chocolates/day	1.0*(1-2)	1.0*(1-2)	.189
Fruits and Vegetables (freq./week)	7.2 ± 6.3	7.5 ± 6.4	.436
Nuts and Pulses (freq./week)	2.0*(1-3)	2.0*(1-3)	.103
Non-Vegetarian foods (freq./week)	4.7 ± 3.3	4.9 ± 3.8	.381
Junk foods (freq./week)	1.3 ± 1.8	1.4 ± 2.1	.252
Carbonated Drinks (freq./week)	2.0* (1-7)	3.0*(1-7)	.146
Sweetened Juices (freq./week)	3.0*(1-7)	3.0* (1-7)	.207
Energy Drinks (freq./week)	0.0* (0-0)	0.0* ± (0-0)	.001***
Fresh Fruit Juices (freq./week)	1.0*(0-3)	1.0*(0-2)	.01***
Physical Activity (freq./week)	4.7 ± 3.0	3.8 ± 3.1	.001**

* Median (25^th^-75^th^ quartiles), ** Independent Samples t-test, *** Mann-Whitney U-Test

**Table 2:** Dietary habits among girls and boys attending SSP marginalized schools, n=1280

**Diet Habits**	**Male n=648** **Mean±SD**	**Female n=634** **Mean±SD**	***P*** ** Value**
Sugar (spoons/day)	2.9 ± 2.8	2.1 ± 2.5	.001 **
Milk (cups/week)	0.0* (0-1)	0.0* (0-1)	.001 ***
Sweets and chocolates/day	1.8 ± 1.8	1.8 ± 1.7	.481
Fruits and Vegetables (freq./week)	7.3 ± 6.8	7.4 ± 5.7	.926
Nuts and Pulses (freq./week)	2.8 ± 2.7	2.4 ± 2.8	.004**
Non-Vegetarian foods (freq./week)	4.9 ± 3.8	4.5 ± 3.2	.030 **
Junk foods (freq./week)	1.0* (0-2)	1.0* (0-1)	.001 ***
Carbonated Drinks (freq./week)	4.5 ± 4.2	2.8 ± 3.2	.001 **
Sweetened Juices (freq./week)	4.0 ± 3.9	3.4 ± 3.1	.001**
Energy Drinks (freq./week)	0.0* (0-1)	0.0* (0-0)	.001 ***
Fresh Fruit Juices (freq./week)	1.0* (0-2)	1.0* (0.5-3)	.091
Physical Activity (freq./week)	4.9 ± 3.1	3.5 ± 2.9	.001**

* Median (25^th^-75^th^ quartiles), ** Independent Samples t-test, *** Mann-Whitney U-Test

### Anthropometric assessment

Senior dental students were trained to measure weight and height of school children on the school visit day. Children’s height was taken with their shoes on and having them stand with their feet together on an uncarpeted floor with the head, shoulders, back and heels placed against a flat wall surface. A manually calibrated scale was used to measure students’ weight. All measurements were taken before students’ lunch break. Measure of children’s weight was obtained in kilograms. Children’s height was measured in centimeters. BMI was calculated by dividing the weight by the height squared and multiplying that total by 10 000. The resultant value was then used to determine where the student fell, percentagewise, among children of the same sex and age group.^27^ Those percentiles were used to determine the child’s weight category as follows: “Underweight”: BMI of less than the fifth percentile; “Normal Weight”: BMI from the fifth percentile to below the 85th percentile; “Overweight”: BMI above the 85th percentile to below the 95th percentile; “Obese”: BMI greater than or equal to the 95th percentile.^28^

Parental informed consents were collected by school administrative staff before the school visits.

### Statistical analysis

All data was recorded using paper sheets and then transferred to an SPSS sheet. Dietary habits were described using proportions and percentages for categorical variables and mean, and standard deviation for continuous variables. To explain variations in dietary habits and BMI, bivariate analysis (Independent samples *t* test) was used to relate these variables with children’s demographic factors (gender and grade), fathers’ employment and mothers’ education. For the sake of the current analysis, father’s employment variable was dichotomized as: regularly employed, irregular employment and unemployed (combined) and mother’s education level was dichotomized to: high school diploma (or less) or beyond high school. All analyses were carried out using the Statistical Package for the Social Sciences (SPSS) version 24 (SPSS Inc. Chicago, IL, USA, 2020)^29^ and significance level was set to 0.05.

## Results

Of the 1308 students who submitted a signed consent form to the school administration, a total of 1282 students completed the questionnaire: a response rate of 98%. Fifty percent of the sample were males and 51.4% were ninth graders. Fifty-one percent of the students had fathers who were unemployed or who had non-regular jobs, 46% of the sample had mothers with lower than a high school education and 34% had mothers with only a high school diploma.

Of our sample, 6% (n = 77 out of 1282) were underweight and 34% (n = 436 out of 1282) were overweight or obese. Among sixth graders, 43% (155 of 360) of the boys and 24% (59 of 247) of the girls were overweight or obese (*P* = 0.001). The opposite was true for ninth graders; 20% (54 of 268) of the boys and 42% (158 of 377) of the girls were overweight or obese (*P* < 0.001).

Sixty-nine percent (n = 888) of the study sample had 3 meals per day, 26.8 % (n = 342) had two and almost 4% (n = 49) had one meal per day. Eighty-five percent (n = 1091) indicated that they snack between meals. Among sixth graders, 51.5% (n = 321) consumed milk daily and among ninth graders, 36.6 % (n = 241) consumed milk daily. Energy drinks were consumed by almost 12% (n = 74) in sixth grade and 20% (n = 132) in ninth grade. Table 1 details the dietary habits among female and male students in the study sample and Table 2 details dietary habits among sixth and ninth graders in the current study sample.

Bivariate analysis showed that ninth graders had more added sugar in their diet than sixth graders (*P* = 0.002), less milk consumption (*P* < 0.001), more energy drink consumption (*P* < 0.001), less fresh juice consumption (*P* < 0.01), and less physical activity (*P* < 0.001) (Table 1).

Consumption of carbonated and sweetened drinks was associated with being overweight or obese (*P* = 0.016, *P* < 0.001).

Consumption of carbonated drinks was higher among children of mothers with a high-school education than among children of mothers with college degrees (*P* < 0.001). In addition, underweight children were associated with mothers educated to high school level or below (*P* = 0.05). Students with fathers who were unemployed or had irregular jobs compared to students with fathers who had regular jobs consumed more junk food (*P* = 0.04),more carbonated drinks (*P* = 0.01) and more sweetened juices (*P* = 0.02*).*

When comparing male and female students, male students consumed more added sugars (*P* < 0.001), more sweetened juices (*P* = 0.001), more junk food (*P* < 0.001), more carbonated drinks (*P* < 0.001), more energy drinks (*P* < 0.001), more non-vegetarian foods (*P* = 0.03), more nuts and legume (*P* = 0.004), more milk consumption (*P* < 0.001) and engaged in more physical activity (*P* < 0.001) (Table 2).

## Discussion

Although many studies addressed dietary habits and nutritional practices among Palestinian children, this study is unique because it focused on students in marginalized schools, mainly located in Area C of the West Bank of the oPt.

The health system in the oPt, in general, is operating under severe pressure because of the occupation, blockade, rapid population growth, dearth of financial resources and scarcities in basic supplies.^30^ In the West Bank, the legislative and physical divisions according to the Oslo Accords have created particularly vulnerable populations in Area C. Of approximately 330 000 Palestinian residents in these areas, 114 000 (35%) have limited access to primary health care services.^31^ In 2017, in response to a question to women living in Area C asking “what specific aid the priority for their households was”,^32^ health infrastructure was the number one priority indicated by almost 25% of the women respondents.^32^ This indicates that health inequality exists with worse health indicators for populations living in Area C, compared to the Palestinian average in the West Bank area.^31^

Health inequality affects access to essential primary and promotional health interventions. These services are necessary for parents and children to promote healthy living, including favorable dietary habits and physical activities. Results in this study suggest that dietary habits worsen between the ages of 12 years and 15 years. Consumption of energy drinks and carbonated drinks increased significantly between sixth and ninth graders. Twenty percent of ninth graders in the current study consumed at least one energy drink per week compared to 14.7% of US adolescents (grades 6-12) in a population-based study.^33^ In addition to negative health side effects of energy drink consumption, likelihood of depression and other behavior problems was associated with higher frequency of energy drink consumption.^33^

Interestingly, results in this study are the first to quantify energy drink consumption among Palestinian adolescents; however, there were a few studies globally^34^ that indicated high consumption of carbonated drinks among this age group, especially boys,^35^ which agrees with the current study’s results.

On the other hand, compared to 44% of adolescents in the current study who consumed milk daily, a study conducted among schoolchildren aged 13-15 years old in public and private schools in the three biggest governorates of the West Bank (Ramallah, Nablus and Hebron) reported only 25% of the study sample consumed milk on a daily basis.^34^ This can be explained by the difference in urbanicity level and related dietary habits between Area C, which is mainly rural, and the urban clusters in the three large cities of Ramallah, Nablus and Hebron. However, in both studies, boys consumed more milk than girls, and in the current study milk consumption decreased significantly between sixth and ninth grades.

In general, boys in the current study’s sample consumed more added sugar and more junk food than girls and consumed as much fruit as girls. However, fruit consumption in both girls and boys needs to be more promoted to reach the level recommended by the WHO of 400 g or five portions per day for this age group.^36^

Regarding physical activity, 45% of the current sample had some sort of physical activity 5 times or more per week compared to 22% in a study conducted in East Jerusalem in the 11 to 16-year-old age group in 2002,^37^ and 20% in a national survey conducted in 2005 among students in grades 6-12. ^26^ In line with the other studies,^26,38^ in Palestine, our results showed that boys are significantly more active than girls. What is interesting, again, is that physical activity decreased significantly between sixth and ninth grades.

When we attempted to explain factors related to favorable dietary habits and physical activity, this study’s results agreed with previous studies on the positive effect of mothers’ education level on normal weight, favorable dietary habits and physical activity.^35,38^

Six percent in this study were underweight compared to 3% in United Nations Relief and Works Agency (UNRWA) schools (which serve only Palestinian refugees in camps) in the same age group (6^th^ and 9^th^ grades),^39^and 4.8% in East Jerusalem in a wider age group (11–16-year-olds).^37^ Twenty-five percent were overweight in this study compared to 12% in UNRWA schools^39^ and 24% in East Jerusalem schools.^37^ Obesity was found in 9% of the current sample compared to 6% in UNRWA schools^39^ and 10% in East Jerusalem.^37^ In general, and according to a global study that pooled data from 200 countries, average obesity in the Middle East, North Africa and the United States was around 20%.^40^

Although this study has a good sampling technique that produced an adequate representative sample of students in marginalized schools according to the SPP and MOE definitions, the data collected was cross-sectional and did not cover all aspects of an essential nutritional evaluation. The bidirectional nature of data collected made confirming associations between demographic characteristics and dietary habits somewhat challenging. However, this study succeeded in describing some important dietary habits and physical activities for this vulnerable population which needs special attention given the political and health conditions under which they suffer.

In conclusion, dietary habits in this sample were not satisfactory compared to health organizations standards; however, they were still better than other parts of the oPt. What is interesting is the disparity in healthy eating and physical activity between boys and girls in this age group and the deterioration of healthy food habits and physical activity between the ages of 12 and 15 (grades 6 and 9). Unfavorable habits are usually related to personal, social, and environmental impediments to accessing healthy food choices and physical activity among this age group.^41^ Lack of awareness, motivation, support and time devoted to practice sports and prepare healthy meals were the main barriers to healthy eating and physical activity among both genders in a cross-country evaluation of seven Arab countries.^41^ In general, girls faced more barriers to physical activity than boys in the seven countries included in the previous study.^41^ In addition to previous identified factors, Palestinian adolescents face extra burdens of occupation, blockade, check points and the weak primary health care system in most of the oPt areas, especially Area C.

In this sample, our results suggest that dietary habits and physical activity worsen between the ages of 12 and 15. Mothers’ level of education is an important factor in being overweight or underweight. Energy drink consumption among ninth graders, and milk consumption and physical activity among girls in general, are two challenges found in this study which need to be addressed urgently and adequately in any health promotional activity targeting marginalized schools in the West Bank area of the oPt. These promotions should start as early as elementary school and focus on ages 12-15 years.

## Acknowledgements

The authors would like to thank Dr. Abdallah Hassan, Mrs. Fidah Musa and dental students at Al-Quds University (Palestine) who contributed to making this work possible.

## Funding

This study received a partial funding from AMIDEAST SSP.

## Competing interests

The authors declare that they have no competing interests.

## Ethical approval

Ethical approval for all aspects of the study was obtained from Al-Quds University Scientific Research Ethics Committee with reference number 32/REC/2018. In addition, an administrative approval to conduct the study in the sampled schools was obtained from the Palestinian Government MOE.

## Authors’ contributions

SA participated in conceptualization, data curation, methodology, writing-original draft; EK is the supervisor of this project and contributed to the methodology, supervision, validation and writing and editing.

## Data Availability Statement

The data that supports the findings of this study are available from the corresponding author upon reasonable request.
